# Treatment of Cardiovascular Disease by Traditional Chinese Medicine against Pregnane X Receptor

**DOI:** 10.1155/2014/950191

**Published:** 2014-06-22

**Authors:** Kuen-Bao Chen, Hsin-Yi Chen, Kuan-Chung Chen, Calvin Yu-Chian Chen

**Affiliations:** ^1^School of Medicine, College of Medicine, China Medical University, Taichung 40402, Taiwan; ^2^Department of Biomedical Informatics, Asia University, Taichung 41354, Taiwan; ^3^Department of Anesthesiology, China Medical University Hospital, Taichung 40447, Taiwan; ^4^School of Pharmacy, China Medical University, Taichung 40402, Taiwan; ^5^Research Center for Chinese Medicine & Acupuncture, China Medical University, Taichung 40402, Taiwan; ^6^Human Genetic Center, Department of Medical Research, China Medical University Hospital, Taichung, Taiwan

## Abstract

Recently, cardiovascular disease, also known as loop circulatory system diseases or disorders, is one of the serious diseases including heart disease, stroke, atherosclerosis, myocardial infarction, hypertension, hypotension, and thrombosis. Human pregnane X receptor, PXR, plays a crucial role in exogenous and endobiotic metabolism for rabbit, rat, mouse, and human. The PXR activation can protect the blood vessels from damage of hazardous substances. In this study we aim to investigate the potent lead compounds as PXR receptor agonist against cardiovascular disease. To improve drug development of TCM compounds, we aim to investigate the potent lead compounds as PXR agonists from the TCM compounds in TCM Database@Taiwan. The top three TCM compounds, bis(4-hydroxybenzyl) ether mono-*β*-D-glucopyranoside (BEMG), ixerisoside, and tangshenoside II, have displayed higher potent binding affinities than the positive control, PNU-142721, in the docking simulation. After MD simulations, which can optimize the result of docking simulation and validate the stability of H-bonds between each ligand and PXR protein under dynamic conditions, top TCM compounds, BEMG and tangshenoside II, maintain most of interactions with PXR protein, which keep the ligand binding stable in the binding domain. Hence, we propose BEMG and tangshenoside II as potential lead compounds for further study in drug development process with the PXR protein.

## 1. Introduction

Recently, cardiovascular disease, also known as loop circulatory system diseases or disorders, is one of the serious diseases including heart disease, stroke, atherosclerosis, myocardial infarction, hypertension, hypotension, and thrombosis. It is the top leading cause of death in the United States and most European countries. More than 83.6 million Americans have the cardiovascular problems; the patients of cardiovascular disease in other Western countries are also growing yearly [1]. Family history, obesity, latent diseases, such as diabetes, gout and kidney disease, and bad habits, diet, the environment of toxic substances, and drugs are the risk factors for cardiovascular disease [2–4]. Environmental pollution and chemicals also promote the occurrence of blood vessel function disorders and cardiovascular diseases. We should consider how to regulate and protect the blood vessels [5].

Nowadays, many distinct mechanisms of diseases have been identified [6, 7] to determine the potential target proteins for drug design against each disease [8–11]. Human pregnane X receptor, PXR, plays a crucial role in exogenous metabolism for rabbit, rat, mouse, and human [2–5]. Some studies indicate that PXR also plays an important role in endobiotic metabolism for rabbit, rat, mouse, and human [12–22]. Activated PXR binds to response elements in the promoters and upregulates the transcription of Phases I and II drug-metabolizing enzymes, for example, glutathione S-transferases (GSTs) and cytochrome P450 (CYP)s, and transporters, for example, multidrug resistance protein 1 (MDR1) [12, 13]. It provides a mechanism for the blood vessels to protect itself and the underlying tissue under exogenous and endobiotic insults [14].

The human pregnane X receptor, PXR (NR1I2, also known as PAR or SXR), is a key transcription factor gene expression and regulation of CYP3A. It is combined by DNA binding domain (DBD) and ligand binding domain (LBD) [19–21]. It is composed of three *α*-helices and five *β*-folds formed around globular ligand binding cavity [22]. PXR can be activated by variant ligands, including drug [12], endogenous compounds [12, 23], and environmental contaminants [24]. PXR has a similar protective effect in the vessel and in liver, which can stop the liquid, the solute, and the cells in the vessel wall. Therefore, the PXR activation can protect the blood vessels from damage of hazardous substances. In this study, we aim to investigate the potent lead compounds as PXR receptor agonist against cardiovascular disease.

Recently,* in silico* researches have been broadly used in the drug design [25–29]. Many compounds extracted from traditional Chinese medicine (TCM) had been determined as potential lead compounds for many different diseases, such as stroke [30–32], tumors [33–36], inflammation [37], metabolic syndrome [38–40], viral infection [41, 42], and some disorders [43–45]. As structural disordered amino acids in the binding domain of protein may affect the ligand binding with target protein and induce side effect [46, 47], the disordered amino acids of PXR protein were predicted before virtual screening. For TCM compounds filtered by virtual screening, the interactions of the docking poses in the docking simulation may be modified under dynamic conditions. We employed the molecular dynamics (MD) simulations to validate the stability of each docking pose. In addition, the biological activities of potential TCM candidates were predicted by three distinct models.

## 2. Materials and Methods

### 2.1. Data Collection

The X-ray crystallography structure of the human pregnane X receptor (PXR) was downloaded from RCSB Protein Data Bank with PDB ID 3R8D [48]. The disordered amino acids of PXR protein were predicted using PONDR-Fit [49] protocol with the sequence of PXR protein from Swiss-Prot (UniProtKB: O75469). The PXR protein has protonated the final structure of protein with Chemistry at HARvard Macromolecular Mechanics (CHARMM) force field [50] and removed crystal water using Prepare Protein module in Discovery Studio 2.5 (DS 2.5). The binding domain was defined by the volume of the cocrystallized anti-HIV drug, PNU-142721. TCM compounds from TCM Database@Taiwan [51] have protonated the final structure and have been filtered by Lipinski et al.'s Rule of Five [52] using Prepare Ligand module in DS 2.5.

### 2.2. Docking Simulation

The prepared TCM compounds have been docked in the binding domain of PXR protein using LigandFit protocol [53] in DS 2.5 which docks ligands into the binding domain using a shape filter and Monte-Carlo ligand conformation generation and then optionally minimized with CHARMM force field [50] and rejected the similar poses by the clustering of saved docking pose. The consensus scores were calculated using the properties of -PLP1, -PLP2, -PMF, -PMF04, dock score, Jain, LigScore1 Dreiding, LigScore2 Dreiding, ligand internal energy, Ludi 1, Ludi 2, and Ludi 3.

### 2.3. Biological Activity Prediction

Three distinct prediction models, multiple linear regression (MLR), support vector machine (SVM), and Bayes network toolbox (BNT) models, were employed to predict the biological activity for the TCM compounds using the pEC_50_ (log(1/EC_50_)) value of 25 compounds out of 33 PXR agonists [54]. The suitable molecular descriptors for constructing the prediction models were selected using genetic function approximation module [55] in DS 2.5, and the protocol estimates the fitness of individual model using square correlation coefficient (*R* ^2^). The prediction models have also been validated by cross validation test. In addition, MLR and BNT models were performed using MATLAB, and SVM model was performed using LibSVM developed by Chang and Lin [56].

### 2.4. Molecular Dynamics (MD) Simulation

For each docking pose in the dock simulation, the protein-ligand complex has been simulated under dynamic conditions with classical molecular dynamics theory using Gromacs 4.5.5 [57]. The topology and parameters for PXR protein with CHARMM27 force field and each ligand were provided using pdb2gmx protocol in Gromacs and SwissParam program [58], respectively. A cubic box is performed with the box edge approximate 1.2 nm from the molecules periphery and solvated using TIP3P water model neutralized by 0.145 M NaCl model using Gromacs. Then the steepest descent [59] was employed to remove bad van der Waals contacts with a maximum of 5,000 steps. In equilibration section, the position-restrained molecular dynamics simulation was employed using linear constraint algorithm, NVT equilibration, Berendsen weak thermal coupling method, and particle mesh Ewald method.

A total of 40 ns production simulation with time step in unit of 2 fs was performed using particle mesh Ewald (PME) option and NPT ensembles. A series of protocols in Gromacs, such as g_rms, g_gyrate, g_msd, g_sas, g_energy, g_rmsf, and do_dssp, was employed to analyze the MD trajectories.

## 3. Results and Discussion

### 3.1. Disordered Protein Prediction

**Figure 1:**
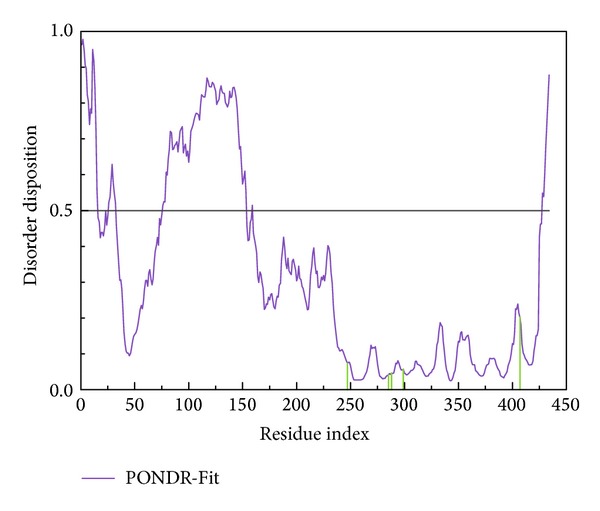
Disordered disposition predicted by PONDR-Fit.

The disordered disposition for the sequence of PXR protein from Swiss-Prot (UniProtKB: O75469) predicted by PONDR-Fit was illustrated in Figure 1. As the residues in the binding domain do not lie in the disordered region, the binding domain of PXR protein has a stable structure in protein folding.

### 3.2. Biological Activity Prediction

GFA (genetic functional analysis) protocol in DS 2.5 was employed with 204 descriptors to determine the ten optimum molecular descriptors for constructing prediction models with 25 compounds of training set. The selected descriptors were ES_Sum_dNH, ES_Sum_ssNH, ES_Sum_sssN, ES_Count_aaCH, ES_Count_ssNH, Num_RingBonds, Molecular_PolarSASA, IAC_Total, Jurs_DPSA_3, and Jurs_PPSA_1. According to these selected descriptors, the functional formula of MLR model was constructed as follows:

(1) pEC50=−1.24629−0.44990×ES_Sum_dNH+1.29360×ES_Sum_ssNH+0.65592×ES_Sum_sssN−0.28544×ES_Count_aaCH−2.05864×ES_Count_ssNH+0.16557×Num_RingBonds+0.01283×Molecular_PolarSASA−0.11968×IAC_Total+0.05874×Jurs_DPSA_3+0.00872×Jurs_PPSA_1.

**Figure 2:**
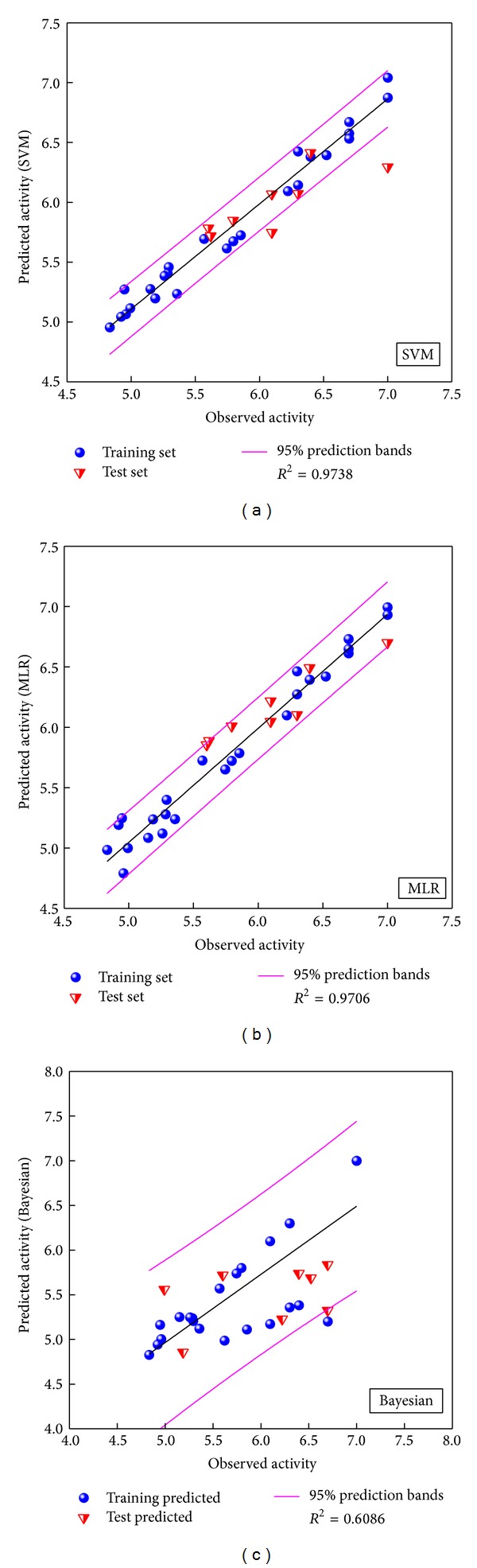
Comparative plots of observed versus predicted activity for (a) SVM, (b) MLR, and (c) BNT models.

The SVM and BNT models were also constructed with the identical training set and descriptors. The correlation of predicted and observed activities shown in Figure 2 illustrates the correlation trend and 95% prediction bands for each prediction model. The square correlation coefficients (*R* ^2^) of training set for SVM, MLR, and BNT models are 0.9738, 0.9706, and 0.6086, respectively. These prediction models are acceptable for predicting activity of PXR protein.

### 3.3. Docking Simulation

**Figure 3:**
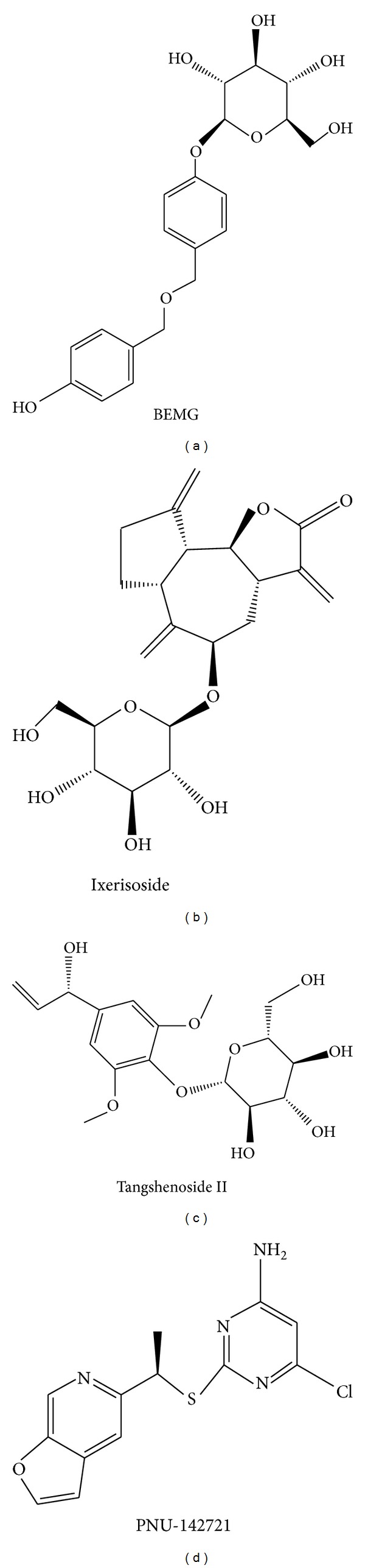
Chemical scaffold of control and the top three candidates: (a) bis(4-hydroxybenzyl) ether mono-beta-D-glucopyranoside (BEMG), (b) ixerisoside, (c) tangshenoside II, and (d) PNU-142721.

**Figure 4:**
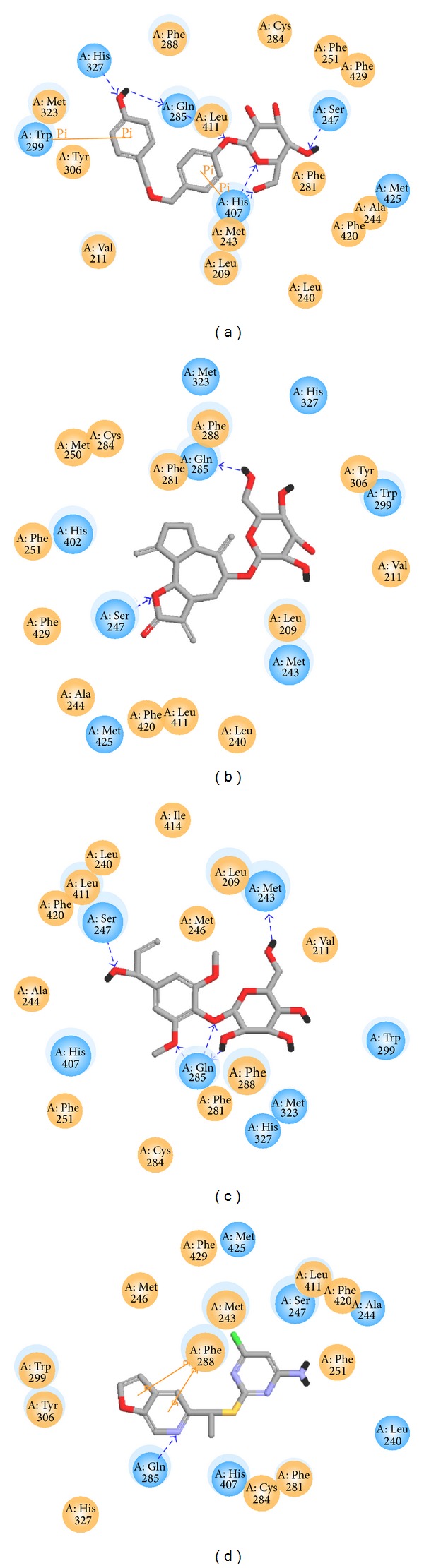
Docking pose of PXR complex with (a) BEMG, (b) ixerisoside, (c) tangshenoside II, and (d) PNU-142721.

**Figure 5:**
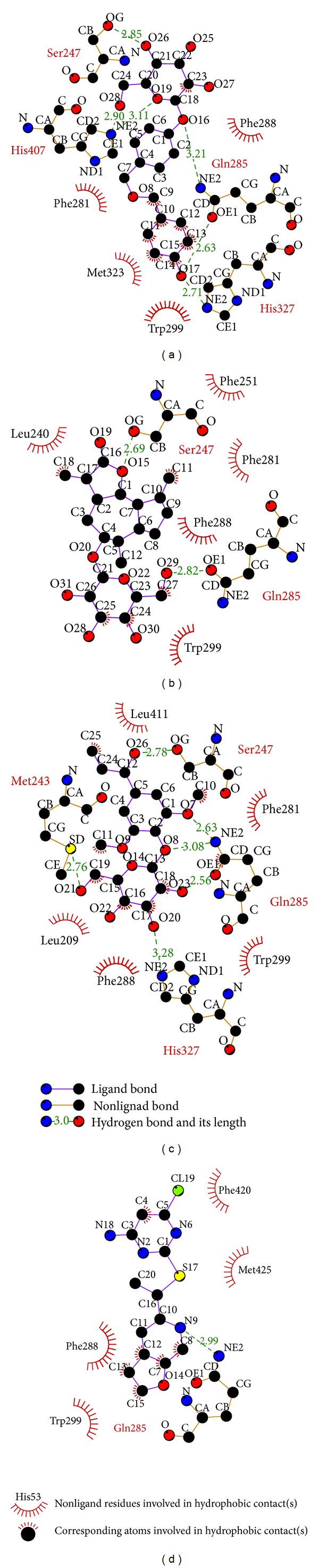
Docking pose of PXR complex with (a) BEMG, (b) ixerisoside, (c) tangshenoside II, and (d) PNU-142721 drawn by LigPlot program.

According to the experimental results (Table 1), the consensus score, dock score, H-bond forming residues, H-bond quantity, and the predicted activities by SVM, MLR, and BNT models are used to rank the top 20 TCM compounds. For the top three TCM compounds, bis(4-hydroxybenzyl) ether mono-*β*-D-glucopyranoside (BEMG), ixerisoside, and tangshenoside II, BEMG was extracted from* Gastrodia elata* [60], which have been indicated the effect of reducing blood pressure, increasing the heart, cerebral blood flow, and reducing cerebral vascular resistance [61, 62]. Ixerisoside was extracted from* Cichorium intybus* [63], which can improve diabetes [64] and clear toxins in the liver [65]. Tangshenoside II was extracted from root of* Codonopsis tangshen* [66], which has excitatory effects for nervous system, and can enhance the body resistance; expansion of peripheral vascular and blood pressure, and inhibit the pressor effect of epinephrine, regulate gastrointestinal motility, anti-ulcer, inhibition of gastric acid secretion, reducing the activity of pepsin, raise leukocyte level declined after chemotherapy and radiation. The chemical scaffold top TCM compounds and PNU-142721 are illustrated in Figure 3. According to the docking poses in Figures 4 and 5, the top three candidate compounds and control have hydrogen bonds (H-bonds) with the common amino acid Gln285 exist. The top three candidate compounds have H-bonds with Ser247. In addition, BEMG still produces hydrogen bonds with His327 and His407 and generates *π* bond with His407 and Trp299. Tangshenoside II will produce additional hydrogen bond with Met243, as well as PNU-142721 will produce *π* bond with Phe288.  Figure 5 illustrates the hydrophobic contacts between each compound and residues in the binding domain. The top three candidate compounds and control have hydrophobic contacts with common residues Phe288 and Trp299, and all TCM compounds have hydrophobic contacts with residue Phe281. The docking results indicate that the top three TCM candidate compounds have higher binding affinities than control. In addition, they have H-bonds with key residues Ser247 and Gln285 and hydrophobic contacts with key residues Trp299 and Phe288.

### 3.4. Molecular Dynamics Simulation

**Figure 6:**
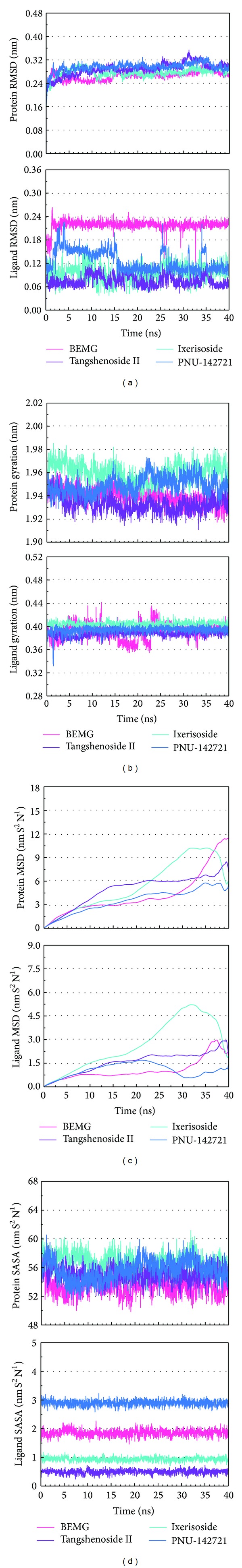
Analysis of MD trajectories generated by Gromacs: (a) root-mean-square deviations (RMSDs), (b) radii of gyration, (c) mean square deviation (MSD), and (d) total solvent accessible surface area (SASA).

**Figure 7:**
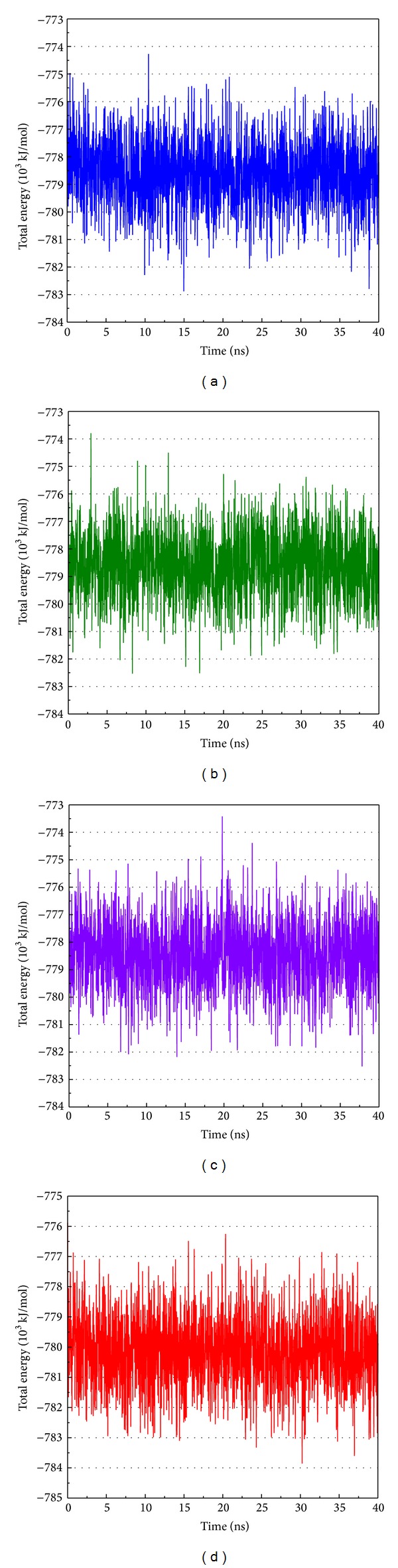
Total energy of PXR complex with (a) BEMG, (b) ixerisoside, (c) tangshenoside II, and (d) PNU-142721.

**Figure 8:**
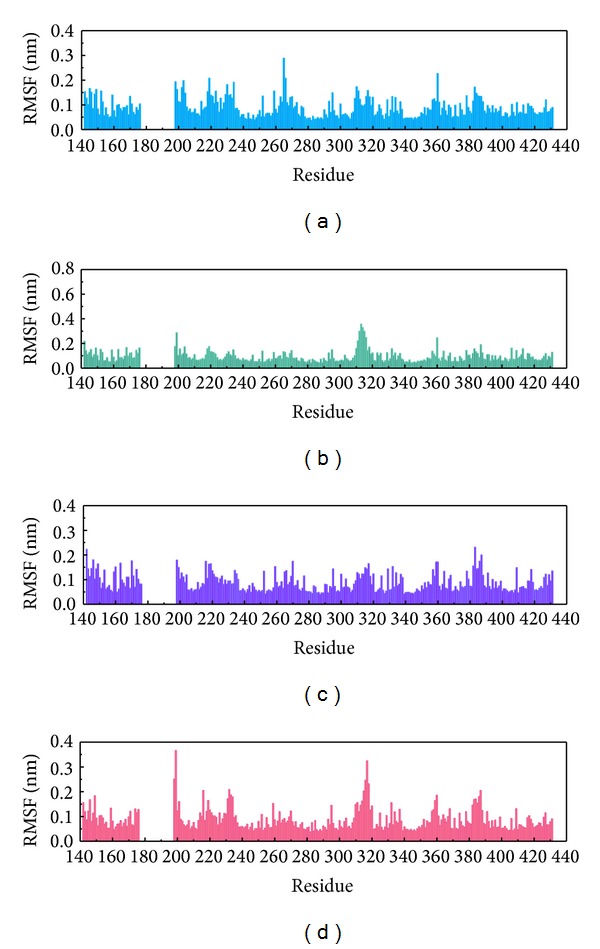
Root mean square fluctuation (RMSF) for residues in PXR complex with (a) BEMG, (b) ixerisoside, (c) tangshenoside II, and (d) PNU-142721 over 35–40 ns MD simulation.

**Figure 9:**
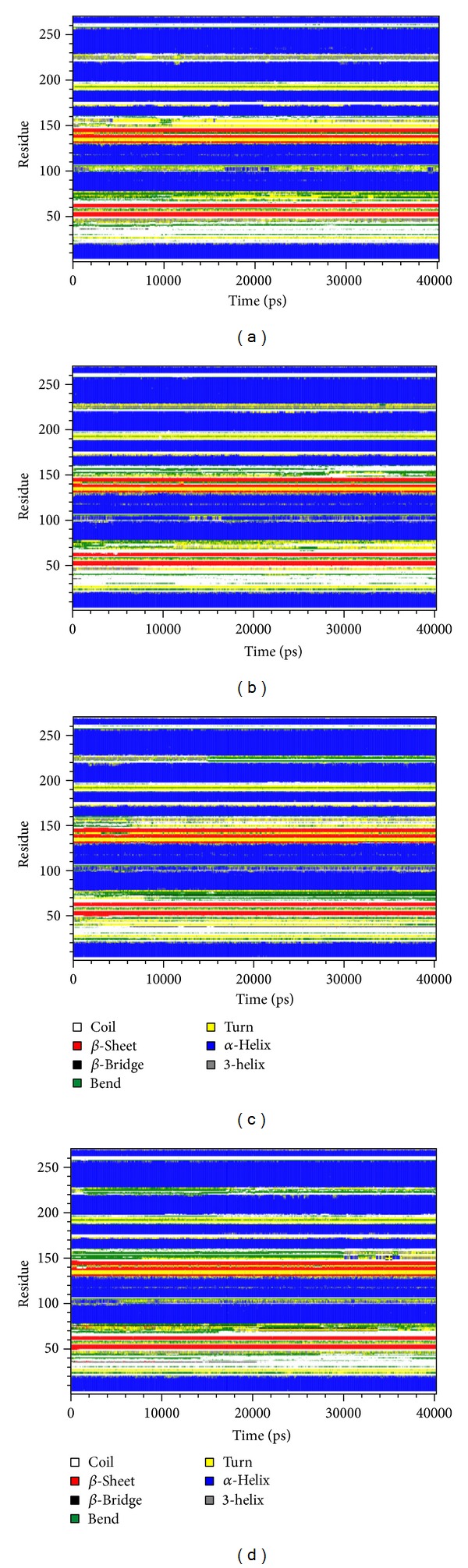
Changes of secondary structure in the PXR complex with (a) BEMG, (b) ixerisoside, (c) tangshenoside II, and (d) PNU-142721 during MD simulation.

MD simulation was employed to validate the stability of interactions between PXR protein and each compound. Root-mean-square deviation (RMSD) illustrated the atomic fluctuations during MD simulation in Figure 6(a). Protein RMSD displays the changes in the protein structure of PXR induced by the TCM candidates and control, which are tended to stabilize after MD simulation. For the ligand RMSD in Figure 6(a), the value of BMEG tends to stabilize after 2 ns of MD simulation at approximately 0.21 nm. For the other TCM candidates and control, the ligand RMSD also tends to stabilize after 20 ns of MD simulation. The variation of radii of gyration for protein and each ligand in Figure 6(b) indicates that each compound may not lead to significant variation to PXR protein under dynamics condition. The slope of the MSD showed in Figure 6(c) indicates that ixerisoside induces larger diffusion changes than others, which has an increase the slope after 20 ns. The variation of solvent accessible surface area (SASA) of PXR protein and each ligand in the complexes over 40 ns of MD simulation is illustrated in Figure 6(d). It shows that there is no significant change in both protein SASA and ligand SASA. The averages of ligand SASA of BEMG, ixerisoside, tangshenoside II, and PNU-142721 are 1.85482 nm/NS^2^, 0.937577 nm/NS^2^, 0.499383 nm/NS^2^, and 2.896435 nm/NS^2^, respectively. For the variation of total energy of each protein complex displayed in Figure 7, there is also no significant change under dynamic conditions.  Figure 8 displays the root mean square fluctuation (RMSF) of each residue in each PXR protein complex. The key residues in docking simulation, which are Ser247, Gln285, Phe288, Trp299, and His407, have less flexibility under dynamic conditions.  Figure 9 displays the change of secondary structure of PXR protein in each complex. There is no significant change in the secondary structure of PXR protein for each protein complex.

**Figure 10:**
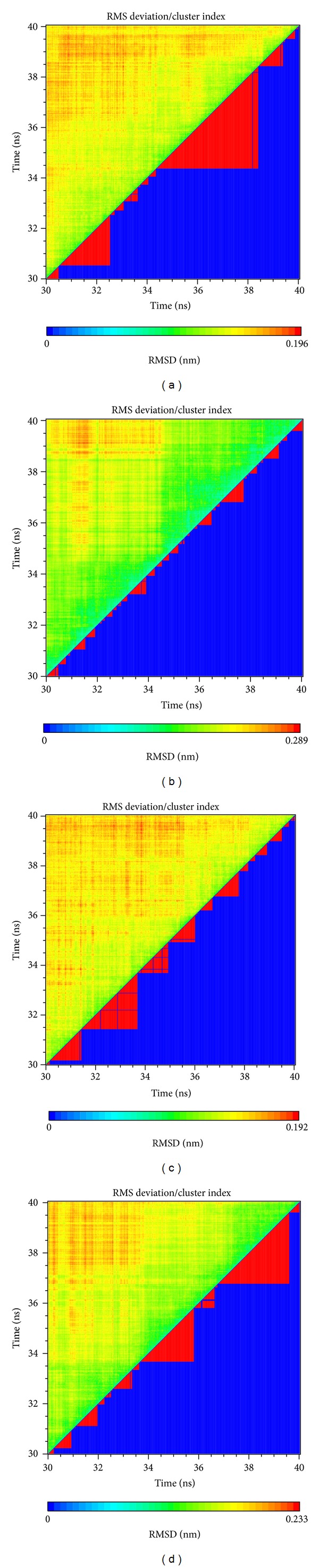
RMSD matrix and clustering diagram of MD conformations over 30–40 ns for PXR complex with (a) BEMG, (b) ixerisoside, (c) tangshenoside II, and (d) PNU-142721. Clusters were calculated using a cutoff of 0.1 nm.

**Figure 11:**
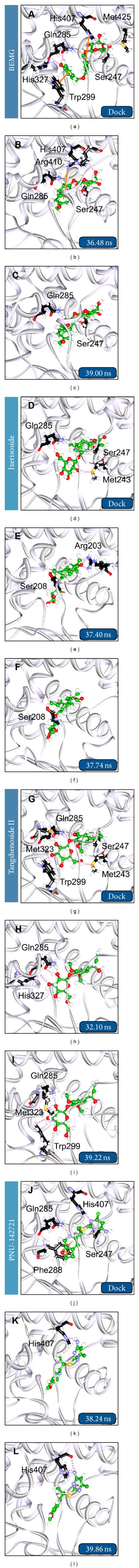
Snapshots of docking pose in docking and MD simulation for PXR complex with (a)–(c) BEMG, (d)–(f) ixerisoside, (g)–(i) tangshenoside II, and (j)–(l) PNU-142721.

**Figure 12:**
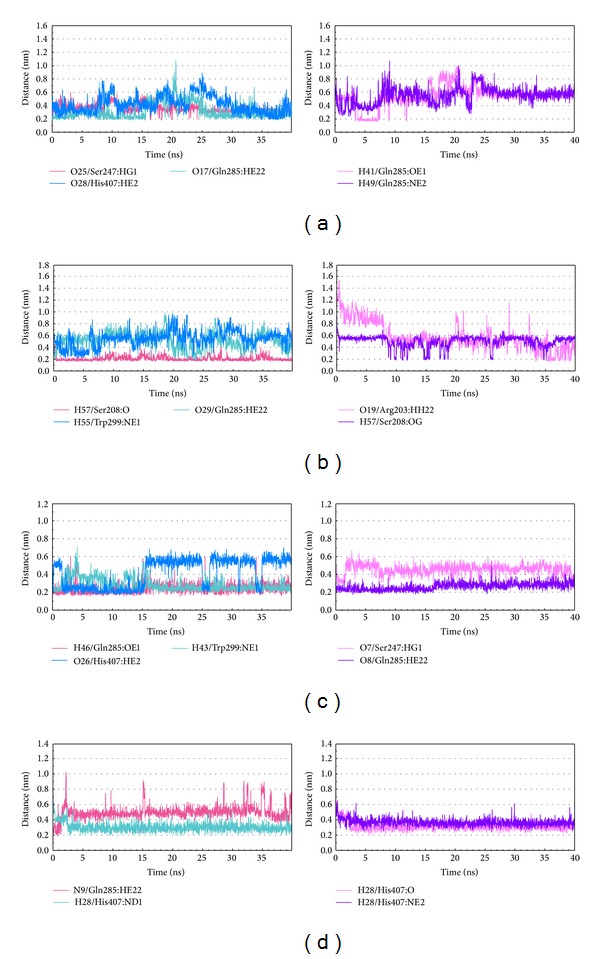
Distance variation of H-bonds for PXR complex with (a) BEMG, (b) ixerisoside, (c) tangshenoside II, and (d) PNU-142721 during MD simulation.

**Figure 13:**
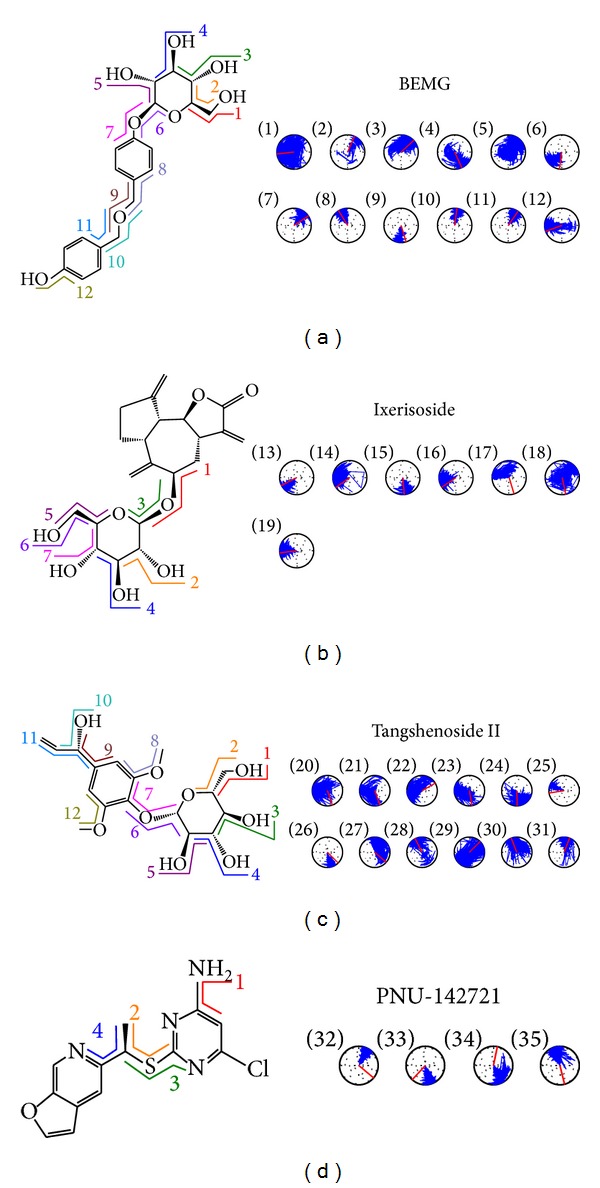
Variation of ligand torsion angles for each PXR complex during 40 ns of MD simulation.

The representative structures of PXR protein complexes after MD simulation were identified by the RMSD values and graphical depiction of the clusters analysis with a RMSD cutoff of 0.1 nm during 30–40 ns of MD simulation (Figure 10). The docking poses in docking simulation and two representative structures after MD simulation for each PXR protein complex are illustrated in Figure 11. For BEMG, it maintains the H-bonds with Gln285 and Ser247. Ixerisoside forms the H-bond with Ser208 instead of the H-bonds in docking simulation. Tangshenoside II also has stable H-bonds with Gln285, Trp299, and Met323 after MD simulation, as PNU-142721 maintains H-bonds with His407. To discuss the stabilities of H-bonds under dynamics condition, the H-bond occupancy for key residues of PXR protein and variation of each H-bond over 40 ns of MD simulation are displayed in Table 2 and Figure 12, respectively. BEMG has the stable H-bonds with Ser247, Gln285, and His407 after 30 ns of MD simulation. Ixerisoside has stable H-bonds with Ser208 and forms an H-bond with Arg203 instead of Gln285 and Trp299. Tangshenoside II has stable H-bonds with Gln285 and Trp299 and loses the H-bond with Ser247 after 2 ns of MD simulation. For control, PNU-142721, has stable H-bonds with His407.  Figure 13 illustrates the variation of torsion angles in each ligand over 40 ns of MD simulation. The variation of each torsion angle supports the result of distance variation of H-bonds in Figure 12.

## 4. Conclusion

This study aims to investigate the potent TCM candidates for PXR protein. The top three TCM compounds, BEMG, ixerisoside, and tangshenoside II, have displayed higher potent binding affinities than the positive control, PNU-142721, in the docking simulation. According to the docking results, the top three candidate compounds and control has hydrophobic contacts with common residues Phe288 and Trp299, and all TCM compounds have hydrophobic contacts with residue Phe281. The docking results indicate that the top three TCM candidate compounds have higher binding affinities than control. In addition, they have H-bonds with key residues Ser247 and Gln285 and hydrophobic contacts with key residues Trp299 and Phe288. The MD simulations are performed to optimize the result of docking simulation and validate the stability of H-bonds between each ligand and PXR protein under dynamic conditions. For the MD simulation, the top three TCM compounds maintain most of interactions with PXR protein, which keep the ligand binding stable in the binding domain. In addition, they have potential bioactivities predicted by the three distinct models. Hence, we propose BEMG and tangshenoside II as potential lead compounds for further study in drug development process with the PXR protein.

## Figures and Tables

**Table 1:** Docking results and predicted pEC_50_ for top TCM compounds and PNU-142721.

Name	CS∗	Dock score	H-bond forming residues	H-bond quantity	Predicted activity∗		
					SVM∗	MLR∗	BNT∗
Bis(4-hydroxybenzyl) ether mono-beta-D-glucopyranoside	11.00	100.59	Ser247, Gln285, His327, His407	6	5.17	2.92	5.29
Ixerisoside	10.00	103.44	Ser247, Gln285	2	6.34	4.22	5.23
Tangshenoside II	8.00	105.556	Ser247, Gln285, Met243	6	6.51	3.77	4.89
Ruine	8.00	104.085	Ser247, His407	4	5.24	2.73	5.22
Crotalaburnine	9.00	100.181	Ser247, Gln285	3	5.62	1.70	4.50
Dihydroferulic acid [3-(4-hydroxy-3-methoxyphenyl) propionic acid]	9.00	98.854	Gln285, His327, Met425	4	6.11	2.89	4.44
Corchoionoside C	8.00	99.075	Ser247, Met425	2	6.36	3.10	4.64
Beta-D-glucosyl-columbianetin	8.00	98.283	Gln285	1	6.05	3.96	5.24
Ethyl rosmarinate	8.00	97.721	Hisa327, His407	2	5.91	2.94	4.63
Persicarin	7.00	102.239	Ser247, Gln285	2	7.24	9.68	5.83
6beta,7beta,16beta,17-Tetrahydroxy-ent-kauranoic acid	7.00	100.892	Ser247, Gln285, His407	5	6.43	4.22	4.96
Androsin	7.00	99.939	Gln285, His327, His407	5	6.35	3.47	4.62
Baihuaqianhuoside	7.00	98.004	Ser247, Gln285, Met243	3	6.08	2.77	4.51
Eleutheroside B	7.00	97.823	Gln285	3	6.58	3.94	4.93
Androsin	6.00	102.747	Gln285, His327, His407	4	6.28	3.22	4.55
4-Hydroxy-3-methoxy-acetophenone-4-O-beta-D-glucopyranoside	6.00	99.008	Gln285, His407	3	6.35	3.47	4.62
Nortrachelogenin	6.00	98.94	Gln285, Met425	2	5.14	1.75	4.64
3-Methoxy-4-beta-D-glucopyranosyloxypropiophenone	5.00	98.645	Ser247, Gln285, His327	4	6.08	2.77	4.51
Azelaic acid	3.00	102.435	Ser247, His327, His407	4	7.16	5.44	3.63
Sulfoorientalol D	3.00	100.402	Gln285, His407	3	6.59	3.90	4.42
PNU-142721∗	0	46.172	Ser247, Gln285, His407	1	5.72	0.54	4.10

PNU-142721: control.

CS: consensus score.

SVM: support vector machine.

MLR: multiple linear regression.

BNT: Bayesian network.

Predicted activity: −log (activity, where activity = % transactivation of PXR receptor × 10 *μ*mol/L).

**Table 2:** H-bond occupancy for key residues of PXR protein complex with the top three candidates and PNU-142721 over 40 ns molecular dynamics simulation.

Ligand	H-bond	Ligand atom	Amino acid	Distance (nm)			Occupancy (%)
				Max.	Min.	Average	
BEMG	1	O25	Ser247:HG1	0.62	0.18	0.36	12.85%
	2	O26	Ser247:HG1	0.90	0.19	0.49	7.60%
	3	O17	Ser247:HG1	0.91	0.18	0.48	4.40%
	4	O27	Ser247:HG1	0.86	0.19	0.47	4.00%
	5	O17	Gln285:HE22	1.08	0.17	0.32	59.90%
	6	O27	Gln285:HE22	0.89	0.18	0.36	31.45%
	7	H41	Gln285:OE1	1.00	0.16	0.54	9.75%
	8	H49	Gln285:NE2	1.07	0.24	0.55	1.95%
	9	O28	His407:HE2	0.89	0.18	0.42	18.95%
	10	O19	His407:HE2	0.88	0.20	0.56	4.20%
	11	O25	His407:HE2	0.61	0.21	0.40	5.75%
	12	H52	His407:ND1	0.78	0.20	0.47	1.80%

Ixerisoside	1	H57	Ser208:O	0.57	0.15	0.21	95.70%
	2	O19	Arg203:HH22	1.55	0.16	0.58	7.15%
	3	H57	Ser208:OG	0.90	0.17	0.50	6.85%
	4	O19	Arg203:HH12	1.66	0.16	0.77	2.45%
	5	O29	Gln285:HE22	0.99	0.18	0.52	6.45%
	6	H55	Gln285:OE1	0.99	0.18	0.60	3.45%
	7	O22	Gln285:HE22	0.91	0.21	0.65	0.50%
	8	H55	Trp299:NE1	0.98	0.21	0.55	5.50%
	9	H56	Trp299:NE1	0.69	0.23	0.45	1.55%

Tangshenoside II	1	H46	Gln285:OE1	0.61	0.15	0.25	84.20%
	2	O8	Gln285:HE22	0.50	0.17	0.26	78.75%
	3	O23	Gln285:HE22	0.51	0.17	0.26	76.70%
	4	O9	Gln285:HE22	0.57	0.20	0.38	13.40%
	5	O7	Gln285:HE22	0.75	0.23	0.47	1.00%
	6	O7	Ser247:HG1	0.67	0.19	0.46	1.10%
	7	O26	Ser247:HG1	0.85	0.19	0.59	0.30%
	8	H43	Trp299:NE1	0.71	0.19	0.28	71.05%
	9	H45	Trp299:NE1	0.69	0.22	0.44	6.50%
	10	O26	His407:HE2	0.70	0.17	0.42	38.05%

PNU-142721	1	N9	Gln285:HE22	1.03	0.17	0.50	2.40%
	2	H28	His407:ND1	0.82	0.19	0.30	54.55%
	3	H28	His407:O	1.05	0.20	0.31	44.55%
	4	H28	His407:NE2	0.65	0.24	0.37	5.60%

H-bond occupancy cutoff: 0.3 nm.
